# Pharyngeal and Cervical Reconstruction and Fistula Management Following Total Laryngectomy: Contemporary Strategies and Evidence

**DOI:** 10.3390/life16091426

**Published:** 2026-08-27

**Authors:** Maximilian George Dindelegan, Alma Aurelia Maniu, Claudia Diana Gherman, Răzvan Alexandru Ciocan, Cosmin Ioan Faur, Alex Victor Orădan, Hunor Levente Horvath, Violeta Necula

**Affiliations:** 1 Department of Otolaryngology, Head and Neck Surgery, Institute of Oncology Prof. Dr. Ion Chiricuță, 400015 Cluj-Napoca, Romania; 2 Department of Surgery-Practical Abilities, “Iuliu Hațieganu” University of Medicine and Pharmacy, Marinescu Street, No. 23, 400337 Cluj-Napoca, Romania; 3 Department of Otorhinolaryngology, “Iuliu Hatieganu” University of Medicine and Pharmacy, 4–6 Clinicilor Street, 400006 Cluj-Napoca, Romania; 4 Department of Surgery II, County Clinical Emergency Hospital, Clinicilor Street, No. 3–5, 400006 Cluj-Napoca, Romania; 5 Faculty of Dental Medicine Alumni, “Iuliu Hațieganu” University of Medicine and Pharmacy, 400347 Cluj-Napoca, Romania; ioan.faur@reginamaria.ro; 6 Regina Maria Dental Department, Regina Maria Private Healthcare Network, 014416 Bucharest, Romania; 7 Department of Plastic and Reconstructive Surgery, Institute of Oncology Prof. Dr. Ion Chiricuță, 400012 Cluj-Napoca, Romania

**Keywords:** total laryngectomy, salvage laryngectomy, pharyngocutaneous fistula, pharyngeal reconstruction

## Abstract

Total laryngectomy (TL) is still the standard treatment for advanced laryngeal and hypopharyngeal cancer, with pharyngeal reconstruction being a challenging determinant of outcomes. We searched PubMed/MEDLINE, Scopus, and Web of Science from inception to July 2026 for studies reporting pharyngeal reconstruction techniques or fistula outcomes after TL. This narrative review synthesizes current evidence on reconstruction following TL across three domains: prevention, reconstruction, and management of pharyngocutaneous fistula (PCF). Defect classification into minimal, partial, and circumferential categories guides reconstructive decision-making, each supported by a distinct evidence stream. Risk stratification identifies prior radiotherapy, poor nutritional status, sarcopenia, and salvage setting as the strongest PCF predictors. For minimal defects, stapler-assisted closure reduces PCF risk in primary TL, and double-layer manual closure outperforms single-layer repair. Partial defects in the salvage setting benefit from vascularized tissue augmentation, with pedicled onlay flaps associated with lower fistula rates than primary closure or free flap patching. For circumferential defects, network meta-analytic data suggest that the free jejunal flap ranks highest for fistula prevention. Comparative cohort data indicate that anterolateral thigh flaps may offer better long-term speech outcomes. The optimal choice depends on the outcome prioritized. Salivary bypass tubes represent the most consistently supported adjunct for fistula and stenosis prevention. PCF management follows a stage-based approach: conservative care for early fistulas, negative pressure wound therapy as a bridge in subacute cases, and vascularized flap reconstruction for chronic or refractory fistulas.

## 1. Introduction

Laryngeal cancer (LC) is the second most common site for head and neck squamous cell carcinoma, after the lip and oral cavity [1]. With the advent of organ-preserving therapies, upfront primary total laryngectomy (TL) is generally reserved for advanced T4a disease. For early-stage LC, the aim is often to preserve the larynx by means of laryngeal-sparing surgeries or definitive radiotherapy (RT). Management of T3 LC typically follows an organ-preserving approach, most commonly concurrent chemoradiotherapy (CRT) or induction chemotherapy followed by either CRT or surgery in case of no response to induction chemotherapy, with salvage TL performed in case of incomplete response. In carefully selected patients, definitive surgical options may include TL, open partial laryngectomy, transoral laser microsurgery (TLM), or transoral robotic surgery (TORS). Although multimodality strategies combining surgery, radiotherapy, and chemotherapy have been used in both laryngeal and hypopharyngeal cancers, surgery remains the cornerstone of treatment, with TL representing the standard procedure for advanced disease [2,3,4].

Across historical evidence, pharyngocutaneous fistulas (PCFs) are the most feared and common early post-operative complication after TL. This complication has a major impact, as it prolongs the length of hospital stay, increases the costs, and most importantly, delays adjuvant therapy and functional rehabilitation [5]. The reported incidence varies widely, with the literature ranging from low single digits to >60%, implying that the risk is highly heterogeneous depending on multiple factors, and the need for risk stratification and standardized perioperative management strategies [6,7].

Numerous systematic reviews and meta-analyses have examined individual reconstructive techniques or reported pooled fistula rates after TL. However, these typically address a single technique or a single endpoint. Few integrate the primary versus salvage setting into a practical decision framework. A clinically oriented synthesis that organizes reconstruction by defect category and connects it to fistula prevention and management is currently lacking. This narrative review therefore aims to (1) synthesize contemporary evidence on pharyngeal reconstruction following TL, organized by defect category; (2) integrate the primary versus salvage distinction into technique selection; and (3) propose a structured, stage-based approach to the prevention and management of PCF.

This is a narrative review based on a structured literature search. We searched PubMed/MEDLINE, Scopus, and Web of Science from inception to July 2026 using combinations of MeSH terms and the following search strategy: (“laryngectomy” OR “total laryngectomy” OR “salvage laryngectomy”) AND (“pharyngeal reconstruction” OR “pharyngocutaneous fistula” OR “pharyngeal fistula” OR “pharyngeal defect” OR “pharyngeal closure” OR “neopharynx”). Reference lists of included articles were hand-searched for additional sources. Additional references were identified and included through reviewer recommendation during peer review. We included original studies of all designs, systematic reviews, and meta-analyses published in English that reported pharyngeal reconstruction techniques and fistula outcomes after TL. Conference abstracts without full-text data, non-English publications, studies focusing exclusively on partial laryngectomy without TL data were excluded. Studies addressing esophageal or tracheal reconstruction without pharyngeal outcomes were also excluded. Study selection was based on the eligibility criteria described above, specifically the publication type, language, focus on TL, and reporting of pharyngeal reconstruction techniques and/or PCF outcomes. Studies meeting these criteria were included in the narrative synthesis. Because this search was not conducted as a systematic screening process, detailed record counts at each stage were not prospectively documented. A total of 84 articles were retained for the present synthesis. As a narrative review, evidence was synthesized qualitatively rather than pooled. A formal risk-of-bias or certainty-of-evidence assessment was not performed; this is acknowledged as a limitation (see Section 7).

## 2. Anatomy and Defect Classification

A functional neopharynx with appropriate caliber, patent and sealed, is the aim of a successful TL. Achieving adequate swallowing and voice rehabilitation represents the goals of a functional rehabilitation. Voice rehabilitation is a demanding objective of TL surgery, typically accomplished by a tracheoesophageal voice prosthesis. However, when prosthetic rehabilitation is unsuitable or unavailable, there are other vocal rehabilitation methods for voice rehabilitation, such as esophageal speech or electrolaryngeal speech.

In order to standardize reconstruction methods upon its goals, it is imperative to classify the pharyngeal defects. A clinical defect classification aligned to the latest comparative studies and meta-analyses is as follows:

### 2.1. Minimal Defect

Studies reported that a pharyngeal wall remnant measuring 1.5 cm relaxed (2.5 cm stretched) may be adequate to prevent dysphagia through primary closure. In this case, the pharyngeal mucosa is sufficient for tension-free primary closure [8,9].

### 2.2. Partial Pharyngeal/Hypopharyngeal Defect

If there is less than 2.5 cm of stretched, non-irradiated pharyngeal mucosa available, or there is insufficient pharyngeal wall to close over a 36-Fr dilator, primary closure is not feasible. These defects require additional tissue augmentation to achieve tension-free closure of the pharynx [9,10].

### 2.3. Circumferential Defect

A circumferential pharyngeal defect is defined as a full thickness defect, involving the entire circumference of the pharyngeal lumen. This results in the complete interruption of the pharyngo–esophageal conduct, requiring complex reconstruction in order to reestablish the alimentary tract continuity [11].

This descriptive defect classification also bears clinical relevance, as each defect category aligns with a different evidence stream. For minimal defects, the data is most robust for primary closure strategy and technical refinements, whereas for circumferential defects the literature most consistently evaluates reconstructive flap choice. In salvage TL, the strongest evidence focuses on prophylactic reinforcement with vascularized tissue to mitigate the risk of PCF [5,10]. The management is widely discussed in Section 4. “Pharyngeal reconstructive options”.

## 3. Risk Stratification and Perioperative Optimization

### Risk Factors and Their Clinical Relevance

One meta-analysis, including 8605 patients, evaluated and quantified 26 different risk factors for PCF. Among the 26 risk factors evaluated, 10 were found to be statistically significant [7]. For clarity, these and other reported predictors can be grouped into four categories: patient-related, nutritional and metabolic, tumor-related, and treatment-related.

Patient-related factors

The development of PCF was related to age (OR 1.29), with patients older than 60 years presenting a higher risk. Smoking was found to significantly increase the risk of PCF (OR 1.62) in patients undergoing TL. Meta-analysis also showed that systemic disease, chronic obstructive pulmonary disease (OR 1.62), and coronary atherosclerotic heart disease (OR 1.82) increased the risk significantly. Diabetes mellitus was mentioned as a risk factor in the literature, but the meta-analysis failed to demonstrate a statistically significant difference [7,12].

Nutritional and metabolic factors

Poor nutritional status is among the strongest modifiable predictors of PCF. Low preoperative serum albumin (OR 2.95) and low hemoglobin (OR 1.97) were both significantly associated with fistula formation [7,13]. In a separate retrospective study of 235 patients, preoperative low skeletal muscle mass (sarcopenia) was independently associated with a higher incidence of PCF (34.9% vs. 20.6%) and a longer hospital stay (median 17 vs. 14 days) [14].

Tumor-related factors

Higher tumor stage (OR 0.81 for T1/T2 vs. T3/T4) and supraglottic localization (OR 0.28 for glottic vs. supraglottic) were associated with a higher incidence of PCF, likely reflecting more extensive resection, reduced residual mucosa, suture–line tension, and impaired vascularization [7,13].

Treatment-related factors

Prior radiotherapy is a consistent and strong predictor of PCF (OR 2.41), impairing local blood circulation and inducing tissue fibrosis [7,13]. Salvage TL is biologically distinct. Prior CRT or RT induces microvascular damage that results in hypovascularized, hypoxic tissues with impaired wound healing [7,15,16]. Salvage TL (OR 1.85) is linked to significantly higher incidence of PCF compared with primary surgery. Furthermore, salvage TL also carries a higher overall complication rate, reported at 67.5% in one meta-analysis [15]. The contribution of individual treatment components remains debated: prior radiotherapy is a robust predictor, whereas preoperative chemotherapy alone has not been shown to increase fistula risk in larger series [7,17].

## 4. Pharyngeal Reconstructive Options

### 4.1. Minimal Defects

In minimal defects that are amenable to primary closure, the key question is closure technique rather than flap selection.

An updated 2026 meta-analysis including 1545 patients compared the efficacy of stapler- assisted versus manual closure of pharyngeal defect following TL. Results showed that stapler-assisted closure offered a statistically significant reduction in the incidence of PCF (RR 0.54). Moreover, the use of a mechanical stapler was associated with both shorter operative time (mean difference of 52.6 min) and decreased hospital stay (mean difference of 3.62 days). However, the subgroup analyses of the same study suggested that PCF rate reduction was significant only in primary TL, and it did not reach statistical significance on salvage TL [18]. Chiesa et al. also reported lower incidence of PCF with stapler-assisted closure compared with manual closure (9.5% vs. 23.4%) [19]. Stapler closure appears to be beneficial in appropriately selected cases, with meaningful advantages in primary TL cases. Ideal candidates have endolaryngeal tumors without hypopharyngeal extension, sufficient non-irradiated pharyngeal mucosa for tension-free stapler application, and anatomy allowing laryngeal delivery without pharyngotomy. Wide liberation of the tracheoesophageal groove and adequate epiglottic retraction are required to allow proper stapler placement. These conditions are most reliably met in primary TL, where the reduction in PCF is statistically significant [5,18,19,20].

Pharyngeal defect closure performed manually can be classified on the basis of the reconstructive technique. Closure may be achieved using either a vertical or a T-shaped configuration, with additional differences concerning the number of suture layers and the type of sutures applied. Horizontal closure seems to be the most advantageous for primary closure, as it combines a wide, neopharyngeal lumen with a lower risk of PCF [21,22]. In contrast, vertical and T-shaped closures appear to have comparable outcomes overall, slightly lower rate of PCF in T-shaped closure, each with distinct advantages and limitations depending on defect anatomy [5,21]. However, horizontal closure is not suitable for all cases, particularly in vertically extended defects.

Regarding the type of suture, studies have compared interrupted versus continuous sutures and revealed a statistically significant decrease in PCF rate when a modified continuous suture was used [23,24]. A comparison of single- versus double-layer pharyngeal closure demonstrated a statistically significant advantage for the double-layer technique, with lower PCF rates (12.5% vs. 33.3%) [25]. Similarly, Saha et al. evaluated conventional two-layer closure against modified techniques incorporating residual constrictor muscle reinforcement and also reported statistically significant findings in favor of standard two-layer closure without muscle reinforcement (0% vs. 27%) [26].

A key limitation of the literature is that suture configuration (vertical vs. T-shaped) is inconsistently defined and often confounded by defect extent. Contemporary syntheses and large cohorts generally fail to demonstrate a consistent PCF advantage for one manual configuration over another, supporting a principle-based approach: tension-free, well-vascularized, appropriately calibrated closure with meticulous mucosal apposition.

### 4.2. Partial Defects and Salvage Reinforcements

Partial pharyngeal defects may require patch augmentation (inlay), interposition of a cutaneous paddle, and/or reinforcement of primary mucosal closure (onlay). The major surgical decision is whether to add vascularized tissue and, if so, whether the goal is reinforcement (onlay) versus replacement (inlay), and with what.

A 2024 multicenter cohort study conducted across 17 centers including 309 patients, evaluated salvage TL with limited defects considered suitable for primary closure. The authors compared primary closure (PC), pedicled flaps (PFs), and free tissue transfer (FTT). They reported PCF rates of 34.5% for PC, 39.1% for PFs, and 22.4% for FTT. On multivariable analysis, both PC and PFs were associated with a significantly higher risk of PCF compared with FTT (RR 2.2 and 2.5, respectively), while FTT conferred a clinical benefit. Long-term functional outcomes at 1 to 2 years were comparable across the three reconstructive strategies [16]. Additionally, a 2022 Bayesian network meta-analysis of salvage TL, comprising 1694 patients, concluded that fresh vascularized tissue transfer can reduce PCF compared with primary closure [27]. A 2024 network meta-analysis of 31 studies further reinforced the value of vascularized tissue in salvage TL, showing lower fistula rates (19% vs. 37%) [28]. Considering these findings, the vascularized flap augmentation is beneficial in salvage cases, although differences in flaps design, indications, and study methodology continue to contribute to the heterogeneity results in the literature.

Bringing well-vascularized tissue can be achieved either locally, through PFs, or FTT. Reconstructive options fall into two categories: pedicled regional flaps and free flaps. Pedicled options include the pectoralis major muscle-based flap, most commonly used, as well as sternocleidomastoid, submental, and internal mammary artery perforator flaps. Free flap options include the temporoparietal fascial, radial forearm, anterolateral thigh, and lateral arm flaps. Tonsbeek et al., in a meta-analysis, compared pectoralis major myocutaneous flap (PMMC), pectoralis major myofascial flap (PMMF), anterolateral thigh free flap (ALTFF), and radial forearm free flap (RFFF). Among these, PMMC showed the highest fistula rate (34%), whereas PMMF (7%) and free flaps had significantly lower rates (ALTFF 10%, RFFF 17%) [10]. Furthermore, a meta-analysis of 1694 patients favored pedicled onlay flaps due to a statistically significant difference between them and all other reconstruction methods, such as free flaps or pedicled flap patches [27]. When comparing reconstructive configurations, pectoralis onlay reinforcement of a primary mucosal closure was associated with a lower incidence of PCF than inlay/interpositional reconstruction (18.2% vs. 40.6%) [29].

Pedicled flap onlay reconstruction was associated with lower PCF rates even compared with free flap onlay and free flap patch techniques [10,27]. A possible explanation is the difference in vascular supply: the pedicled pectoralis major flap is nourished by vessels arising from a non-irradiated donor area, whereas free flaps are typically anastomosed to cervical vessels that may have been affected by prior radiotherapy. Mueller et al. reported that radiotherapy-related extracellular matrix remodeling and fibrosis in the recipient bed may impair healing and increase free flap complications [30]. By contrast, when patch reconstruction is required because primary closure is not feasible without tension, free flap patching may be more effective than pedicled patching.

Beyond microvascular and pectoralis-based options, the supraclavicular artery flap (SCAF) has been reported as a reliable pedicled alternative for partial pharyngeal reconstruction. In a comparative study of 77 laryngopharyngectomy reconstructions, the SCAF achieved complication rates comparable to the RFFF and ALTFF [31]. Its skin paddle is thin and pliable. The pedicle arises outside the typical irradiated field and donor-site morbidity is low. These characteristics make the SCAF particularly useful for patch augmentation of partial defects when a free flap is not feasible or when a vessel-depleted neck limits microvascular options [32].

### 4.3. Circumferential Defects

Circumferential defects after TL require whole pharyngeal tube reconstruction. Early local methods, such as the Wookey flap, required multistage procedures and carried considerable morbidity [33]. Subsequently, pedicled and free myocutaneous or fasciocutaneous flaps, along with the free jejunal flap, became the main reconstructive options [34,35,36]. Myocutaneous and fasciocutaneous flaps may be configured either as a tube, by suturing the opposite edges together to form a neopharyngeal conduit, or in a U-shaped fashion, by anchoring the lateral edges to the prevertebral fascia [37,38].

A large network meta-analysis compared five techniques of pharyngeal reconstruction (tubed PMMC, tubed ALTFF, tubed RFFF, free jejunal flap [FJF], U-shaped PMMC). Among the evaluated reconstructive techniques, FJF showed the lowest PCF risk (10%) and the highest probability of being the optimal strategy for fistula prevention. On the Contrary, t-PMMCF was associated with the highest PCF incidence (42%), whereas t-ALTFF, U-shaped PMMCF, and t-RFFF showed intermediate risks (23%, 25%, 27%). Regarding neopharyngeal stenosis, U-shaped PMMCF appeared to perform best, with the lowest absolute risk (11%), while t-PMMCF again showed the least favorable profile (27%) [11].

### 4.4. Functional Outcomes

Nevertheless, PCF represents only one dimension of reconstructive success. When long-term functional outcomes are also considered, the hierarchy of the most predictable flap may shift. Long-term function determines the patient’s actual quality of life.

Swallowing, speech, and stricture

These outcomes may diverge even when fistula rates are comparable. In a 2023 comparative cohort, ALTFF and FJF achieved identical fistula rates (5% each), but differed in functional outcomes. FJF showed lower stricture rates (5% vs. 21%), while ALTFF achieved significantly better speech (83% vs. 28%) [39]. Among circumferential reconstructions, U-shaped PMMCF showed the lowest stenosis risk (11%) and t-PMMC the highest (27%) [11].

Dilation burden

Pharyngoesophageal stenosis requiring dilation is common and thought to be underreported. In one TL cohort, 34% of patients required at least one dilation within a year and 60% over the full follow-up. Free flaps generally required fewer dilations than pedicled myocutaneous reconstructions, attributed to their greater pliability and larger lumen diameter [40].

Time to oral intake

Reported intervals vary widely across studies and by surgical reconstruction technique, the data being heterogeneous, which precludes direct comparison. In salvage series after ALTFF reconstruction, mean resumption of oral intake was 15 days [41]. In pedicled reconstructions, median intervals were 23 days after PMMC versus 15 days for PMMF [42].

In a comparative series, long-term enteral feeding was required in 11.4% of cases after ALTFF reconstruction versus 3.9% after FJF. Free flaps outperformed pedicled flaps in overall feeding-tube dependence [40].

Flap failure and donor-site morbidity

Free tissue transfer is reliable in experienced centers, with flap loss occurring in less than 2% of ALTFF pharyngoesophageal reconstructions. However free flaps require a donor-site, which has its associated morbidities. Thigh donor sites can be closed primarily in approximately 90% of ALTFF reconstructions, but wound complications, sensory changes, and functional deficits at the harvest site remain a problem [43]. Pedicled flaps avoid microvascular anastomosis. Nonetheless, they carry their own donor-site complications. In a systematic-review of 1751 pectoralis major flaps, the pooled donor-site complication rate was 4% [42].

Hospital stay

Length of hospital stay is reported inconsistently and varies both by technique and complication status. Mean stay ranged from 9 days after ALTFF reconstruction to a median of 29 days in another free-flap series and rose substantially when complications occurred [43,44]. These large variations reflect heterogeneous protocols and inconsistent data reporting across studies, rather than a true technique-related effect, which limits cross-study comparison.

Quality of life

Patients reconstructed with free flaps reported significantly better dysphagia-specific quality of life scores than those with primary closure or pedicled flaps. Data suggests that reconstructive choice has a measurable impact on patient-reported outcomes beyond objective measures [44]. PCF is a recognized cause of delay in adjuvant RT [45]. However, how different reconstructive techniques influence the incidence of treatment delay has not been systematically evaluated as a standalone endpoint in comparative reconstructive studies.

Overall, reconstruction of circumferential defects should be guided not by a single endpoint, but by a balance between fistula prevention, stenosis risk, swallowing recovery, and speech outcomes. When feasible and supported by local expertise, FJF remains a strong default option, particularly because of its favorable PCF profile. When jejunal transfer is contraindicated or abdominal surgery is undesirable, U-shaped PMMC may represent the most suitable alternative among pedicled techniques, whereas tubed fasciocutaneous free flaps remain valuable options when functional priorities—particularly speech and swallowing—favor their use.

## 5. Adjuvants and Perioperative Optimization

Nutritional and metabolic status are among the strongest modifiable predictors of PCF [7,13,14]. Optimization should therefore begin before surgery. Low preoperative albumin (<3.5 g/dL) and low hemoglobin are consistent predictors of fistula formation [13]. Preoperative correction of these deficits is advisable. In one series, no patient developed PCF after targeted nutritional evaluation and support, compared with a baseline fistula rate of 34.8% [46].

Sarcopenia deserves specific attention. Low skeletal muscle is independently associated with both higher PCF rates and longer hospital stay. Preoperative protein supplementation and structured exercise programs can improve muscle mass and functional status before surgery, thus reducing PCF rates [14].

Smoking cessation should be started as early as possible. A meta-analysis found that at least four weeks of preoperative cessation lowers wound complications by approximately one third [47]. These measures are inexpensive and low risk. They complement, rather than replace, the intraoperative and reconstructive decisions.

Beyond flap selection and closure design, several adjunctive perioperative measures may further influence outcomes after TL and pharyngeal reconstruction. Among these, salivary bypass tubes (SBTs) represent one of the few interventions supported by relatively consistent meta-analytic evidence. In one retrospective cohort study, SBT was associated with significantly lower PCF rates compared to no SBTs (25% vs. 64.3%; OR 0.185). Furthermore, SBT was linked with a lower proportion of prolonged fistulas requiring more than 20 days to close (36.4% vs. 83.3%; OR 0.114), and earlier resumption of oral intake among patients who developed PCF, with a median advantage of approximately 16.5 days [48]. One 2022 systematic review and meta-analysis on 1960 patients, evaluating SBT placement, reported a pooled PCF incidence of 15.8% in the SBT group and demonstrated significantly lower odds of PCF compared with patients managed without SBT (OR 0.40). Similarly, the pooled incidence of pharyngeal stenosis in the SBT group was 12.3%, with SBT use also associated with reduced odds of stenosis (OR 0.43) [49]. Overall, these findings suggest that intraoperative SBT placement may help prevent both PCF and pharyngeal stenosis.

Enhanced Recovery After Surgery (ERAS) pathways may also contribute to improved perioperative outcomes, even though laryngectomy-specific protocols remain heterogeneous. In this context, ERAS Society recommendations for major head and neck cancer surgery with free flap reconstruction provide evidence-based measures—including nutritional optimization, antibiotic prophylaxis, thromboprophylaxis, multimodal analgesia, goal-directed fluid therapy, and early mobilization—that are likely relevant to wound healing, complication reduction, and length of stay in complex laryngectomy reconstructions [50].

Perioperative acid suppression has also been proposed as an adjunctive measure. Gastroesophageal and laryngopharyngeal reflux may expose the pharyngeal suture line to acid and pepsin, potentially impairing mucosal healing. In a prospective, double-blind, placebo-controlled randomized trial, 14 days of perioperative enteral omeprazole significantly reduced the incidence of PCF after primary TL, from 6 of 19 patients (31.6%) in the placebo group to 1 of 21 patients (4.8%) in the treatment group [51]. Although based on a single small trial, this suggests that proton pump inhibitors may represent a low-risk adjunct supporting suture-line healing, warranting further evaluation.

In addition, tracheoesophageal puncture (TEP) timing may be considered an adjunctive decision in high-risk settings. A 2018 systematic review and meta-analysis comparing primary and secondary TEP found no significant differences in voice outcomes, but suggested a higher PCF risk with primary TEP, thereby supporting consideration of secondary TEP in patients with an elevated baseline fistula risk, such as those undergoing salvage surgery or reconstruction in heavily irradiated fields [52]. This issue remains debated. More recent cohort data indicate that primary TEP can be performed safely, including in irradiated patients and in those undergoing enlarged laryngectomy with pectoralis major reconstruction, with a reported PCF rate of 5.9% and no significant association between primary TEP and complications [53]. Similarly, in a comparison of primary-fit TEP between primary and salvage laryngectomy, fistula rates did not differ significantly between groups [54]. The optimal timing therefore remains individualized, balancing earlier voice rehabilitation against the perceived fistula risk in a given patient.

## 6. Management of PCF

PCF is typically diagnosed within the first postoperative 7–11 days, but the reported timing is heterogeneous in the literature depending on surveillance protocols and the definition used, with some salvage cohorts describing a later median onset [55,56]. Definitions vary across the literature. Some studies record only clinically evident, skin-communicating fistulas. Others also include contained leaks detected on contrast swallow. Reported PCF rates should therefore be interpreted against the definition used and the surveillance protocol applied. Although, no gold-standard test for early diagnosis exists, fever in the early postoperative period has been identified as a useful clinical predictor of subsequent PCF development [57,58,59].

### 6.1. Conservative Management

Management of PCF begins with assessment of fistula severity, local tissue condition, and the likelihood of spontaneous closure. Approximately 60–80% of fistulas may heal with conservative measures only [60]. Although most fistulas heal with conservative measures, prolonged healing by secondary intention has been associated in some series with subsequent pharyngoesophageal stricture, likely reflecting circumferential scarring of the healing tract [61]. Nonetheless, patients managed conservatively—particularly those with prolonged healing—warrant longer-term surveillance of swallowing function and stricture formation. Escalation to surgery remains frequent in the salvage setting. In a multicenter salvage cohort, 57% of PCFs resolved with wound care alone and 43% required operative treatment [55]. This heterogeneity highlights the lack of standardized treatment protocols and the importance of timely reassessment and appropriate escalation.

Standard conservative care includes broad-spectrum antibiotic therapy, along with compressive wound care, and enteral nutrition via a naso-gastric feeding tube [45]. Beyond the standard conservative care, several non-surgical adjunctive strategies have been described for selected cases of PCF. The supporting evidence remains limited.

A systematic review by Tandara et al. reported an overall closure rate of 92.6% and a mean healing time of 25 days with approaches such as botulinum toxin injection, scopolamine patch, hyperbaric oxygen therapy (HBOT), and negative pressure wound therapy (NPWT). However, these data derive largely from small, uncontrolled descriptive series and do not allow clear identification of ideal candidates [59]. HBOT is biologically attractive because repeated hyperoxic exposure may promote wound healing through angiogenic and reparative mechanisms, including VEGF-related pathways [62]. Published clinical evidence for HBOT in PCF remains sparse. Practical concerns also persist, including cost-effectiveness and a theoretical risk of stimulating tumor growth [63].

Similarly, negative pressure-based techniques, including endoluminal vacuum approaches, have shown encouraging results in small case series, with reported closure rates of 83–92% [64]. NPWT is emerging as a promising adjunct in the management of PCF, but the literature is still controversial. Its use in the cervical region is technically challenging because the proximity of the tracheostoma and local secretions may compromise the maintenance of an effective seal [65]. Despite these limitations, NPWT appears to be well tolerated, with limited reported side effects, and its overall costs may be comparable to conventional wound care through fewer dressing changes and shorter hospital stay [66,67].

Botulinum toxin injection offers a different strategy by temporarily reducing salivary flow, thereby limiting one of the main factors contributing to fistula persistence. However, its use in this setting remains off-label and is supported only by very limited clinical experience [68].

Despite these promising findings, the available evidence is weakened by the absence of control groups, limited sample sizes, and poor adjustment for major confounders such as prior radiotherapy and comorbidity burden. Accordingly, these approaches are best regarded as selective adjuncts—particularly in heavily irradiated or surgically hostile fields—rather than substitutes for definitive surgical repair.

### 6.2. Surgical Management

Conservative treatment remains the initial approach for most PCFs. No universally accepted guideline defines the optimal timing of surgery or the ideal reconstructive technique. Conservative management is less effective in salvage and irradiated settings. Fistula healing with conservative care was achieved in 80% of cases after primary TL, compared with 54% after salvage TL [69]. Earlier surgical intervention may therefore be warranted in high-risk patients.

Escalation to operative management is indicated for major or poorly healing fistulas. Specific triggers include failure of conservative treatment, exposed great vessels, flap compromise or necrosis, deep neck infection or abscess, persistent high-output salivary leakage, and unfavorable local tissue conditions [70]. Additional factors lower the threshold for surgery: late presentation, large fistula size, peristomal or mid-neck location, and severely irradiated, infected, indurated, or poorly vascularized tissue [71,72]. PCF management may be guided by wound stage: early fistulas are usually treated conservatively, subacute fistulas may benefit from NPWT as a bridge strategy, and chronic non-healing fistulas, persisting beyond 3 months, generally require definitive surgical reconstruction [73,74].

In small fistulas with favorable, non-irradiated local tissue, direct closure can be an option as first step, however, high recurrence rate, especially in irradiated patients is a key limitation [75,76].

The basic principle of surgical repair is to introduce healthy, well-vascularized tissue into the compromised field. A retrospective cohort study, including 177 patients, suggested that local flap repair had limited durability, with high recurrence rates (35.3%) and frequent need for subsequent regional flap reconstruction [76]. By contrast, more recent series support pedicled pectoralis major flaps, either as a muscle or myocutaneous flap, and pedicled deltopectoral (DP) flaps, as a pragmatic choice for surgical repair of PCF. The pectoralis major flap remains the most commonly used regional option, offering high closure rates, up to 94% in some studies [77].

The DP flap is another reliable pedicled option. Its advantages include robust vascularity, straightforward dissection, avoidance of microvascular anastomosis, and shorter operating time. The donor site lies outside the irradiated field, which allows transfer of healthy tissue and explains the low rate of fistula recurrence [78].

The SCAF has gained renewed interest in the repair of PCF, because it is particularly advantageous in scarred, poorly vascularized necks, where identifying the recipient vessels for free-flap reconstruction may be difficult. Its thin and pliable fasciocutaneous design allows easier inset than bulkier PMMC or DP flaps and avoids muscle-atrophy-related strictures [70,79].

In many cases, regional pedicled flaps remain preferable to free flaps because they provide reliable vascularized tissue without the technical demands and recipient–vessel limitations of secondary microsurgery in a scarred neck. Nevertheless, FTT remains indispensable in more complex scenarios, especially in cases of extensive mucosal loss, failure of prior regional repair, hostile irradiated tissues, or when completion pharyngectomy with tubular reconstruction becomes necessary [9,76,77]. Its main advantage is the transfer of well-vascularized tissue from outside of the irradiated or infected field.

Among FTT options, RFFF has been most frequently used because of its reliability, ease of harvest, and low donor–site morbidity. However, the largest reported series suggests that free-skin-flap reconstruction using RFFF alone may not be sufficient, as closure was achieved in only half of cases. Closure rate increased substantially after additional procedures, such as a second free flap, supplementary local flaps, or concurrent pedicled pectoralis reinforcement. These findings support the value of combining free-flap lining with pedicled external reinforcement [80].

Jejunal free flap has been used in extensive or circumferential defects, as it provides a thin, pliable, well-vascularized tissue, and it appears to be relatively resistant to fistulization. However, it often requires an additional pedicled flap for skin closure, thus increasing the operation time, and it also introduces abdominal donor–site morbidity with the harvesting of the intestinal flap [76,81].

The ALTFF represents another attractive option due to its versatility and ability to be used for reconstructing large composite defects using a single flap [70,80]. Iteld et al. used multi-island ALTFF reconstruction, using the transverse cervical vessels for anastomosis, with successful repair achieved in all cases [82]. These features make the ALTFF a particularly useful option when both lining and external repair are required, especially in irradiated, vessel-depleted necks.

A novel, minimally invasive endoscopic approach has been recently described, based on transoral closure of the fistula tract by reapproximating the platysma skin flap to the pharyngeal defect. In the reported series, all fistulas achieved successful closure, suggesting that it may be an effective approach in selected cases. However, its applicability is limited to small fistulas with preserved platysma coverage and adequate transoral accessibility [83].

Regardless of flap choice, one consistent technical principle is the double-layered closure, combining viable local tissue and an additional vascularized flap for external reinforcement. This approach avoids placing suture lines and tension breakpoints on the same plane, helps obliterate dead space, and may reduce the risk of recurrence [84].

## 7. Discussion and Limitations

The evidence synthesized in this review varies in strength and consistency across the domains addressed. Recognizing where the evidence is robust and where it is fragile is essential for interpreting the proposed framework.

Some of the findings rest on relatively consistent evidence. Prior RT and the salvage setting are consistently associated with a higher risk of PCF [7,15,16]. The benefit of SBTs in reducing fistula and stenosis is supported by pooled analyses [48,49]. Stapler-assisted closure reduces PCF in primary TL [18,19], and double-layer closure outperforms single-layer repair [25,26].

Other areas rest on weaker foundations. The comparative ranking of free and pedicled flaps derives largely from retrospective series and network meta-analyses, not randomized clinical trials [16,27,28]. Functional and patient centered outcomes—swallowing, speech, and quality of life—are inconsistently reported [39]. This limits direct comparison and precludes firm ranking of techniques on these endpoints. Adjuncts, such as NPWT and HBOT remain promising but are supported mainly by small series [63,64].

Taken together, the evidence supports a defect-based, stage-adapted approach as a reasonable organizing principle. Specific technique rankings should be therefore interpreted with caution, given the absence of head-to-head randomized comparisons.

This review has several limitations. First, it is a narrative rather than a systematic review. The literature search was structured and reproducible, but a formal risk-of-bias or certainty-of-evidence assessment was not performed. Second, the evidence base is dominated by retrospective, single-institution studies. Definitions of PCF are heterogeneous, outcome reporting is inconsistent, and follow-up varies. Third, publication, and language bias cannot be excluded, as only English-language publications were included. Fourth, the proposed frameworks have not been prospectively validated, and their impact on clinical outcomes remains untested.

These limitations define the focus for future research. Standardized definitions of PCF and uniform outcome reporting are needed to allow meaningful comparisons across studies. Comparative reconstructive studies should be conducted with clearly defined defect categories, particularly in the salvage setting. These studies should also extend beyond fistula rates and include swallowing, voice, and quality-of-life outcomes.

## 8. Conclusions and Recommendations

Pharyngeal reconstruction after total laryngectomy lacks a unified decision-making framework. Techniques are frequently selected based on institutional familiarity rather than on a structured assessment of defect size, tissue quality, and patient-specific risk. This review synthesizes the available evidence into a defect-driven, stage-adapted approach intended to support reconstructive planning and fistula management. This framework is derived from predominantly observational and heterogeneous evidence and is proposed as a decision-support tool rather than a validated standard; prospective validation is warranted.

The central principle is that defect size dictates the reconstructive strategy and must be anticipated preoperatively, not discovered intraoperatively. Preoperative imaging and endoscopic assessment allow the surgeon to classify the expected defect as minimal, partial, or circumferential, to select the appropriate reconstructive method, and to plan donor sites before the patient enters the operating room. The recommended strategy for each defect category and clinical setting is summarized in Table 1.

For minimal defects, primary closure is adequate. For partial defects, vascularized tissue augmentation reduces fistula rates. If primary mucosal closure is achievable, a pedicled onlay flap over the suture line is the most effective strategy. If primary closure is not feasible, free flap patching performs better than pedicled patching.

Among perioperative adjuncts, salivary bypass tubes carry the most consistent structured framework and should be incorporated into high-risk reconstructions as standard practice.

**Figure 1:**
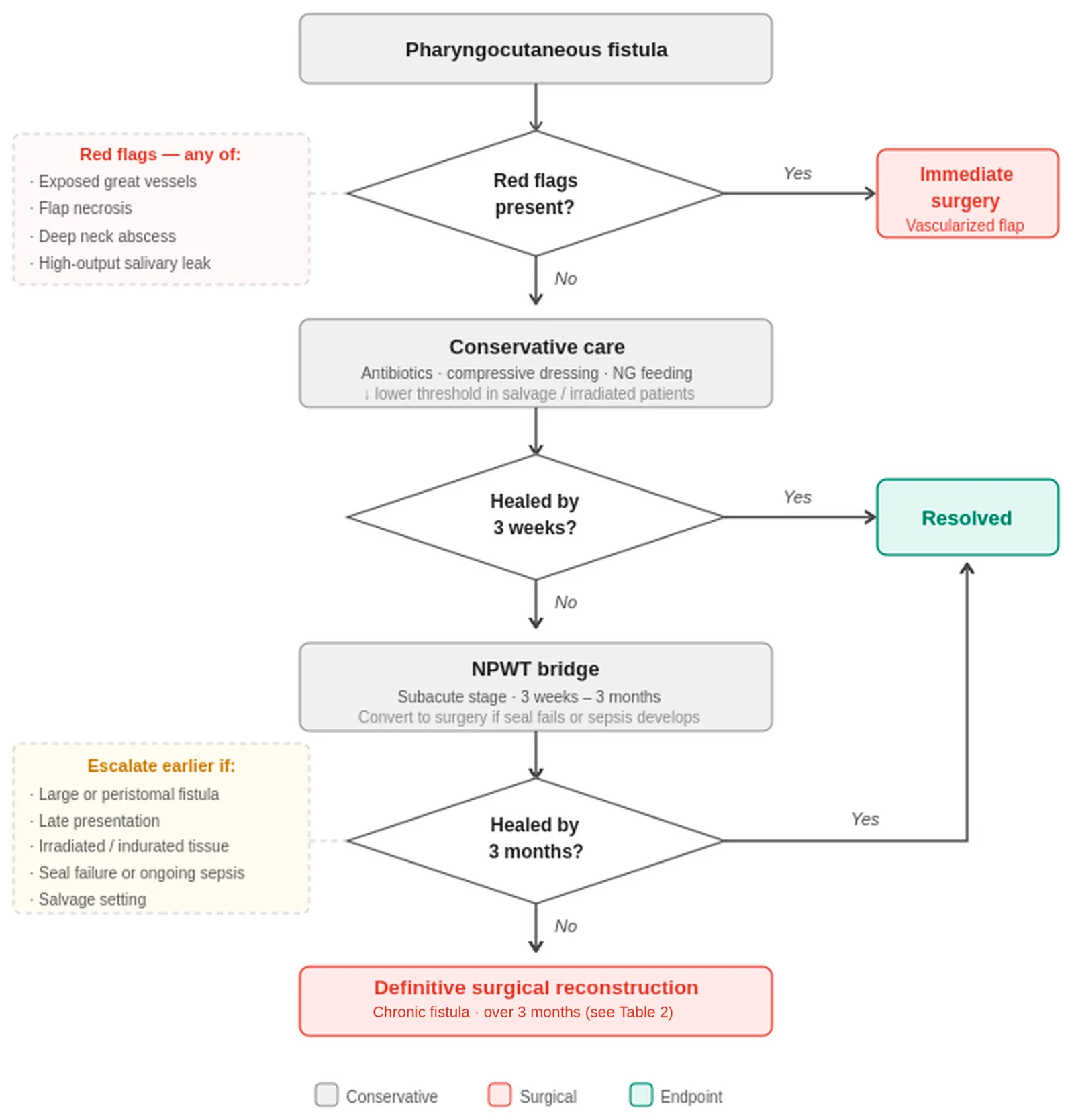
Stage-based algorithm for the management of pharyngocutaneous fistula after total laryngectomy. Abbreviations: NG, nasogastric; NPWT, negative pressure wound therapy.

Fistula management requires a structured, stage-based approach rather than prolonged empirical observation (Figure 1). The key principle is timely escalation: early fistulas are managed conservatively, subacute fistulas benefit from negative pressure wound therapy as a bridge, and chronic fistulas persisting beyond three months will not heal spontaneously and require vascularized flap reconstruction. Red flags—exposed great vessels, flap necrosis, deep neck abscess, or high-output salivary leakage—mandate immediate surgery regardless of stage. In salvage and irradiated patients, the threshold for surgical escalation should be lowered, as conservative management is substantially less effective in these populations.

When surgery is indicated, flap selection should follow the condition of the recipient neck rather than a fixed institutional preference. The guiding principles are straightforward: pedicled flaps when the irradiated field compromises recipient vessels, the SCAF when the neck is scarred and vessel-depleted, and free tissue transfer when regional options have failed or mucosal loss is extensive. Specific decision rules by clinical scenario are provided in Table 2.

Meaningful progress in this field will require standardization of PCF definitions and severity grading, prospective risk-stratified comparative studies, and the inclusion of swallowing, voice, and quality-of-life endpoints alongside fistula rates. Until such data are available, the framework proposed in this review offers a structured foundation for clinical decision-making.

## Figures and Tables

**Table 1:** Reconstructive strategy of the pharynx after total laryngectomy by defect category and clinical setting.

Defect	Setting	Recommended Strategy	Supporting Evidence	Ref.
Minimal	Primary TL	Stapler-assisted closure in selected cases; otherwise, manual double-layer closure	Stapler vs. manual: PCF RR 0.54; operative time −52.6 min; LOS −3.62 d. Separate cohort: PCF 9.5% vs. 23.4%	[18,19]
Minimal	Salvage TL	Manual double-layer closure. Stapler benefit not established in this setting	Stapler subgroup did not reach significance in salvage [18]. Double- vs. single-layer: PCF 12.5% vs. 33.3% [25]. Two-layer without constrictor reinforcement: 0% vs. 27% [26]	[18,25,26]
Minimal	Salvage TL with elevated PCF risk	Consider prophylactic vascularized onlay augmentation over the closure	FTT 22.4% vs. PC 34.5% vs. PF 39.1%; RR 2.2 (PC) and 2.5 (PF) vs. FTT [17]. Vascularized transfer reduces PCF vs. primary closure [27]; 19% vs. 37% across 31 studies [28]	[16,27,28]
Partial	Salvage/irradiated Mucosal edges can be approximated	Mucosal suture reinforced with a pedicled myofascial onlay flap (PMMF) over the suture line	Onlay vs. inlay/interpositional: PCF 18.2% vs. 40.6% [29]. PMMF 7% vs. PMMC 34% [10]. Pedicled onlay superior to free flap onlay and patch [10,27]	[10,27,29]
Partial	Salvage/irradiated Mucosal edges cannot be approximated	Free-flap patch (ALT or radial forearm)	ALTFF 10%, RFFF 17% vs. PMMC 34% [10]. Where patch is required, free flap patching may outperform pedicled patching	[10]
Circumferential	Fistula prevention prioritized	Free jejunal flap	Lowest PCF (10%) and highest probability of optimal fistula prevention in network meta-analysis. Tubed PMMC 42%; t-ALTFF 23%; U-PMMC 25%; t-RFFF 27%	[11]
Circumferential	Speech rehabilitation prioritized	Tubed ALTFF	Identical PCF (5% each); intelligible speech 83% vs. 28% favoring ALTFF; stricture 21% vs. 5% favoring FJF	[39]
Circumferential	Jejunal transfer contraindicated or abdominal surgery undesirable	U-shaped PMMC	Lowest neopharyngeal stenosis among five compared techniques (11% vs. 27% for tubed PMMC); PCF 25%	[11]
Any high-risk reconstruction	Salvage, irradiated, or circumferential defect	Add intraoperative salivary bypass tube	Pooled PCF 15.8% with SBT; OR 0.40 vs. no SBT. Pooled stenosis 12.3%; OR 0.43 [39]. Retrospective cohort: PCF 25% vs. 64.3% (OR 0.185); prolonged fistula 36.4% vs. 83.3% (OR 0.114); oral intake resumed ~16.5 d earlier [38]	[48,49]

Total laryngectomy (TL); pharyngocutaneous fistula (PCF); length of stay (LOS); free-tissue transfer (FTT); primary closure (PC); pedicled flap (PF); anterolateral thigh free flap (ALTFF); pectoralis major myocutaneous flap (PMMC); pectoralis major myofascial flap (PMMF); free jejunal flap (FJF); radial-forearm free flap (RFFF); salivary bypass tube (SBT); t-, tubed configuration; U-, U-shaped configuration.

**Table 2:** Flap selection for surgical PCF repair.

Clinical Scenario	Preferred Option	Rationale	Ref.
Small fistula, non-irradiated, favorable tissue	Direct closure	Simplest option; local repair recurs in 35.3%, predominantly in irradiated patients	[75,76]
Standard salvage repair	Pectoralis major flap (PMMF or PMMC)	Most commonly used regional option; pedicle arises outside the irradiated field; closure rates up to 94%	[77]
Non-irradiated donor site; operative time a constraint	Deltopectoral flap	Robust vascularity, straightforward dissection, no microanastomosis; donor site outside irradiated field explains low fistula recurrence	[78]
Scarred, vessel-depleted neck; no usable recipient vessels	Supraclavicular artery flap	Thin and pliable; easier inset than bulkier PMMC or DP; avoids muscle-atrophy strictures; no recipient vessels needed	[70,79]
Extensive mucosal loss, prior regional flap failure, or hostile irradiated field	Free tissue transfer (RFFF or ALTFF) + pedicled external reinforcement	RFFF alone achieved closure in only ~50% of cases; closure improved substantially with additional pedicled reinforcement	[76,80]
Circumferential defect requiring completion pharyngectomy	Free jejunal flap + pedicled flap for external cover	Thin, pliable, relatively fistula-resistant; often requires an additional pedicled flap for skin closure	[76,81]
Composite defect requiring both lining and external cover	Multi-island ALTFF	Single flap supplies both layers; transverse cervical vessels remain usable in irradiated necks	[70,80,82]

Pectoralis major myocutaneous flap (PMMC); pectoralis major myofascial flap (PMMF); radial forearm free flap (RFFF); anterolateral thigh free flap (ALTFF).

## Data Availability

No new data were created or analyzed in this study. Data sharing is not applicable to this article.
